# Dislocation response of ECC-RC composite supporting structures of tunnels passing through active fault

**DOI:** 10.1038/s41598-024-65523-1

**Published:** 2024-07-02

**Authors:** Shunguo Wang, Zude Ding, Chenghua Shi, Haibing Cai, Yusheng Chen, Wenyun Ding, Juan Huang

**Affiliations:** 1 Faculty of Civil Engineering and Mechanics, Kunming University of Science and Technology, Kunming, 650500 China; 2 School of Civil Engineering, Central South University, Changsha, 410004 China; 3 School of Civil Engineering and Architecture, Anhui University of Science and Technology, Huainan, 232001 China; 4 Kunming Survey, Design and Research Institute Co., Ltd. of China Railway Second Institute, Kunming, 650200 China

**Keywords:** Engineered cementitious composite (ECC), Composite supporting structure, Fault dislocation response, Model tests, Finite element analysis, Civil engineering, Natural hazards

## Abstract

To address the problems of the conventional composite supporting structures (CCSSs) such as insufficient anti-dislocation performance and deformation capacity, this study used Engineered Cementitious Composite (ECC) lining sections instead of the traditional lining sections and optimized support design parameters, resulting in the development of novel ECC-RC composite supporting structures (ECSSs) of tunnels passing through active fault. The dislocation response characteristics and their parameter sensitivity of the ECSS was revealed by way of 1/25-scale fault dislocation model tests and finite element analysis. The test results show that the mechanical response characteristics and the failure modes of the CCSS and the ECSS are similar under reverse fault dislocation. Compared with the CCSS, the anti-dislocation performance of the ECSS is significantly improved by introducing of the ECC lining and optimizing the design parameters. The vertical deformation of the ECSS and the range of influence under the same dislocation are significantly decreased, and the strain are reduced to different degrees. This phenomenon shows that by improving the material properties, shortening the spacing of aseismatic joints and optimising the thickness of the shock absorption layer, the stress conditions and applicability under deformation of the structure are improved. The ECSS benefits from the crack resistance and toughening effect of fibres, the degree and scope of cracking of the ECSS are significantly reduced compared with those of the CCSS, and internal and external through cracks and local spalling are absent. The results of finite element analysis show that the overall damage degree of the ECSS is decreased and the damage range is increased by decreasing the strength of the surrounding rock in the fault zone. The fault dislocation response pattern of the ECSS varies depending on the fault type. The damage degree caused by different fault types follows the order of normal fault, strike-slip fault, and reverse fault from large to small. However, the damage range caused by the strike-slip fault is significantly larger compared to normal fault and reverse fault. In the design of fault resistance, the surrounding rock conditions of the fault zone and the form of fault dislocation should be considered.

## Introduction

Active faults of different scales are widely distributed in China, especially in Western China, where fault activity is frequent^1^. With the promotion of infrastructure construction projects in the western region, such as the Sichuan–Tibet railway and the central Yunnan water-diversion project, a large number of tunnel projects are undergoing construction, and the number of tunnels crossing active fault zones is increasing. For example, the Ya'an-Linzhi section of the Sichuan–Tibet railway passes through eight active fault zones in the form of tunnels^2,3^. The Xianglushan tunnel of the water-diversion project in Central Yunnan passes through the Lijiang–Jianchuan fault, Heqing–Eryuan fault and other active Holocene faults^4,5^. Earthquake damage investigation shows that active faults in high-intensity earthquake areas pose a great threat to the safety of tunnel structures^6,7^. Fault dislocation is the main factor that causes the seismic damage of tunnel structures crossing active faults^8–10^.

In recent years, scholars have conducted in-depth research on the impact of tunnels crossing active faults, exploring the response characteristics and damage mechanisms of tunnels under fault displacement^11–13^. Furthermore, scholars have successively studied fault dislocation prevention and control measures, such as strengthening surrounding rocks; articulated designs and buffer reservation designs based on expanding excavation and setting up shock absorption layers, to reduce the tunnel damage caused by fault dislocation and discussed the supporting structure fortification system of segmentation, zoning and grading^14–17^. Theoretical analyses, numerical calculations, model tests and other methods are adopted to study the mechanical response characteristics and failure mechanism of the fault dislocation of tunnel structures in combination with the supporting structure systems of cross-fault tunnels^18–27^.

The above research on the fault resistance of tunnel structures focused on the structural level, setting a single shock absorption layer, aseismatic joints, flexible joints or a combination of several anti-dislocation measures. It cannot be ignored that no matter what measures are taken, it is difficult to avoid the damage of the typical parts of the lining, and the damage of the lining is still significantly serious in the dislocation area, and the existing research pays less attention to the improvement of the fault resistance performance of the lining segment. Due to tunnel lining susceptible to dislocation and collapse under strong earthquakes across active faults, improving the lining performance and damage tolerance in fault dislocation areas is of great importance for preventing and controlling lining collapse. Engineered Cementitious Composite (ECC) material is a new structural material that exhibits high strength, high toughness, and high damage resistance. It demonstrates notable strain hardening properties under to tensile, bending, and shear loads. ECC can significantly improve the safety and deformation adaptability of the structure, showing high ductility characteristics^28–32^. The results of previous model tests and numerical simulation analysis demonstrated that ECC linings have significantly better ductility, deformation and bearing capacity than the traditional lining, as well as excellent fault resistance and shock absorption performance^33,34^.

However, experimental studies on the anti-dislocation of ECC composite supporting structure currently do not exist, and the lack of experimental data limits the field application and promotion of these structures. Therefore, this paper propose ECC-RC combined support structures (ECSSs) for tunnels passing through active faults with advantages of the ultra-high toughness and high damage resistance of ECC materials. The ECSS replaced some of the reinforced concrete lining sections with steel-reinforced ECC lining and optimised the support design parameters. In this paper, the fault dislocation response model tests of the ECSS and the CCSS were carried out by the research and development of a 1/25-scale multifunctional fault dislocation device, and studied the mechanical response and damage characteristics of the two structures under reverse fault dislocation. Further studied the parameters sensitivity of the dislocation response of the ECSS by the finite element model of dislocation mechanical response, and provided scientific bases for evaluating the potential application of new material combination structures in fault-resistant and seismic mitigation of tunnels passing through active faults.

## Experimental design

### Project overview

**Figure 1:**
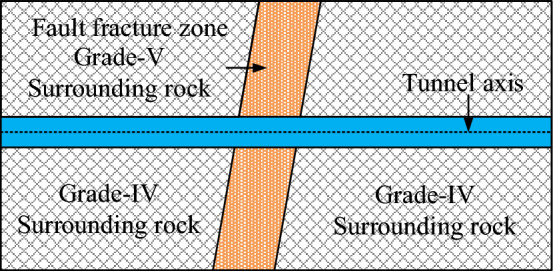
Sketch of geological profile.

The model test in this paper is based on the Renhe Tunnel, which is a highway tunnel in Yunnan province, China. The Renhe Tunnel is 3225 m long, and its maximum buried depth is 368 m. This tunnel crosses an active reverse fault, which is a branch of the Xiaojiang Fault Zone, with the fault dip of 80° and the fault width of 30 m. The surrounding rocks in the fault area are strongly weathered Cambrian limestone and Devonian dolomite, which are grade-V surrounding rocks. The rock mass of the hanging wall is moderately weathered shale and sandstone and that of the footwall is moderately weathered sandstone and dolomite. Both of these rock masses are grade-IV surrounding rocks, as shown Fig. 1. The tunnel section crossing the active fault adopts the expanded excavation and articulated design to reduce the effects of fault dislocation. The shock absorption layer is set along the circumferential direction of the tunnel, and the spacing aseismatic joint is set along the longitudinal direction.

### Overall test scheme

**Figure 2:**
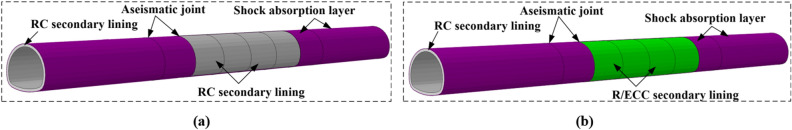
The design of CCSS and ECSS. (**a**) Conventional composite supporting structure (CCSS), (**b**) ECC-RC composite supporting structure (ECSS).

Two types of composite support structures are used in the model tests. The first type is based on the design of the supporting structure of the Renhe Tunnel crossing the active fault zone. The specific parameters are as follows: the lining is lined with C30 reinforced concrete, and its thickness is 60 cm. The shock absorption layer is set along the lining, and its thickness is 5 cm. The aseismatic joints are arranged in the longitudinal direction, and their spacing is 12 m (that is, the conventional composite supporting structure, hereafter referred to as CCSS, corresponding to test condition 1). The section of the support structure is expanded by 40 cm relative to the ordinary section. The second structure maintains the ordinary section type without expansion, and the R/ECC is used to replace the conventional C30 reinforced concrete within the fault zone. The parameters of the shock absorption layer and the aseismatic joints are optimised in accordance with previous research results. The thickness of the shock absorption layer is 10 cm, and the joint spacing is 9 m (that is, ECC-RC composite supporting structure, hereafter referred to as ECSS, corresponding to test condition 2), the design of the two composite support structures are shown in Fig. 2. The composite structural parameters in the model test are shown in Table 1.

**Table 1:** Test scheme.

Test conditions	Supporting structure type	Lining section	Spacing of the aseismatic joint (m)	Thickness of the shock absorption layer (cm)	Tunnel lining materials
Condition 1	CCSS	Section with expansion (SF5f.)	12	5	RC
Condition 2	ECSS	Section without expansion (SF5e)	9	10	R/ECC and RC

**Figure 3:**
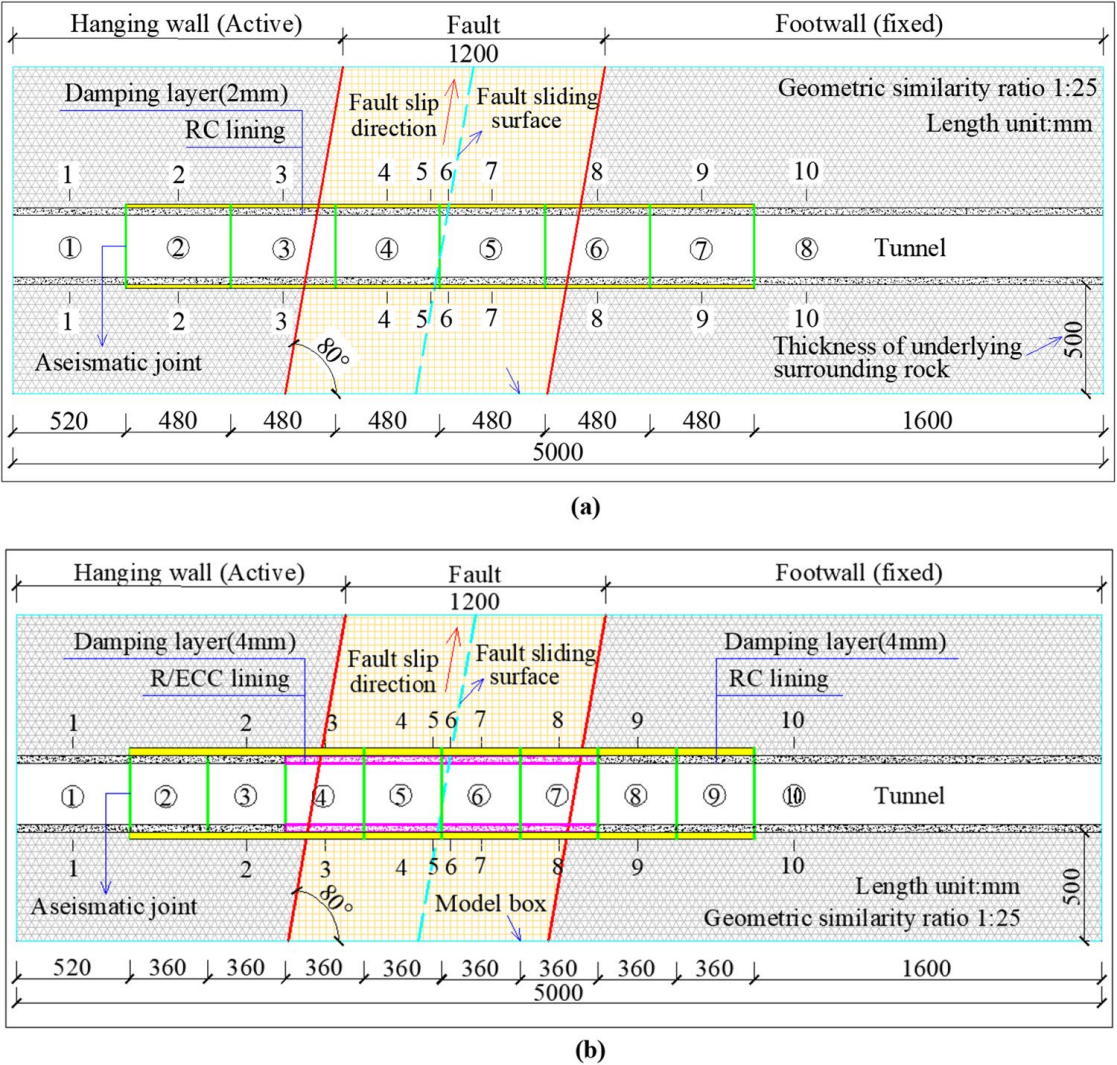
Design of the fault dislocation simulation test of the tunnel structure. (**a**) Model test condition 1 (CCSS), (**b**) Model test condition 2 (ECSS)**.**

In the model test, the buried depth of the tunnel is considered as 1.36 m (that of the corresponding prototype is 34 m), the width of the fault zone is 1.2 m (that of the corresponding prototype is 1.2 m), the fault dip angle is 80° and the sliding surface is located in the middle of the fault fracture zone. Given that the secondary lining is the main bearing structure of the operating tunnel and due to the limitations of the geometric scale of the test, only the secondary lining is considered in the tunnel structure in this test^21^. For test conditions 1 and 2, 8 and 10 lining segments are set, respectively. The dislocation simulation test design of the two composite supporting structures is shown in Fig. 3. In the model test, the right side of the model box is fixed (the foot wall of the fault is a fixed plate), and a loading device is installed at the bottom of the left side to lift the left box upward along the 80° inclination (the hanging wall of the fault is a movable plate) to simulate the reverse fault stick slip dislocation.

### Experimental system

**Figure 4:**
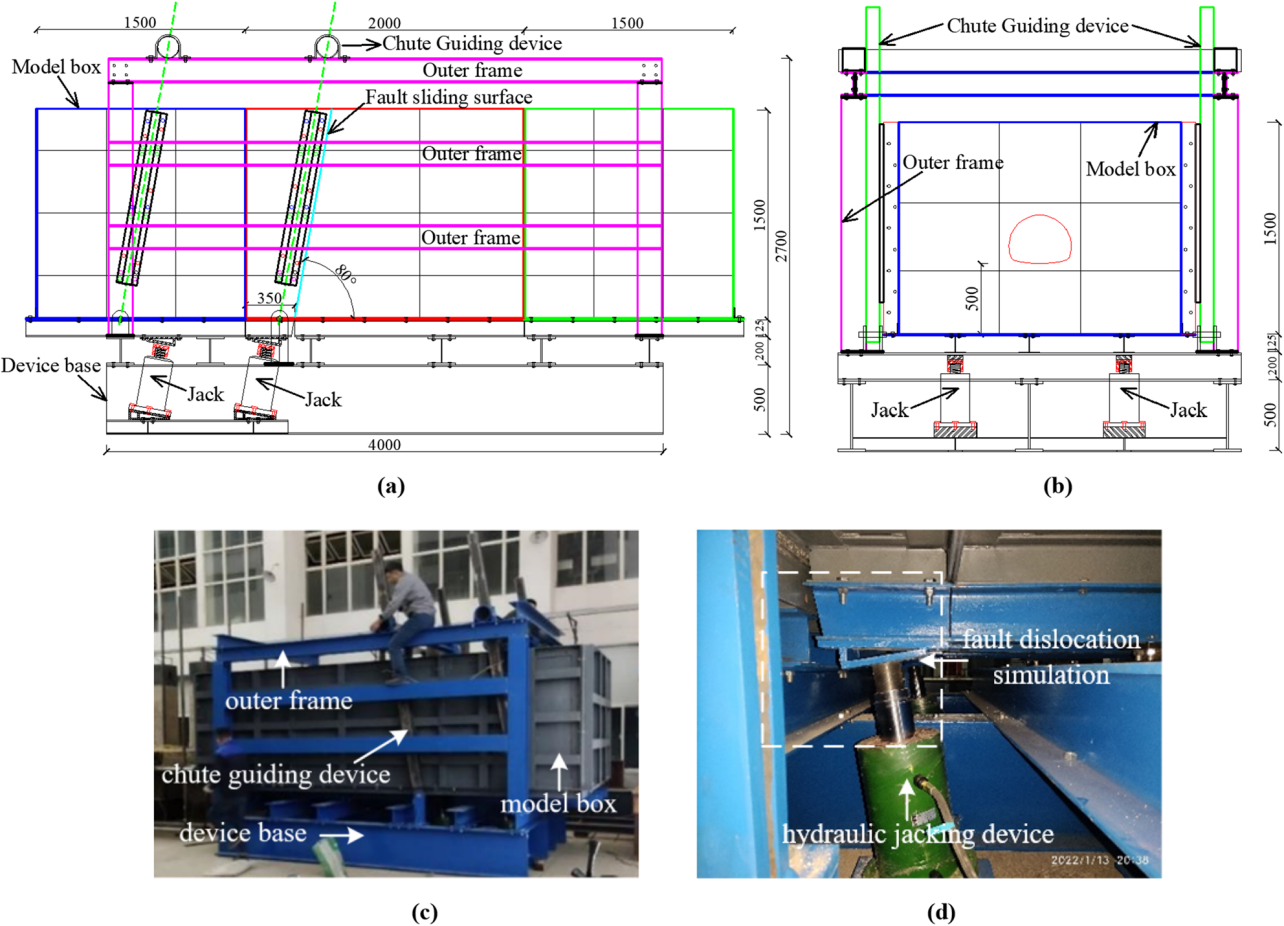
Diagram of fault dislocation simulation loading test device. (**a**) Front view, (**b**) Side view, (**c**) Fault dislocation simulation loading system, (**d**) Hydraulic jacking device.

Currently, most research on fault rupture testing utilizes small-scale tests. However, these tests often have several limitations. Firstly, due to the small scale of the test, there is a high requirement for the preparation of similar materials. Although the test process allows for the observation of general patterns in tunnel structure damage, it does not accurately quantify the damage effect. Additionally, small-scale model tests struggle to consider the effects of fault boundary conditions and gravity, resulting in noticeable deficiencies. Therefore, an independent fault rupture loading model test system was developed to study the damage characteristics of tunnel structures under the influence of stick–slip reverse fault. The system is composed of a model box, a base, an outer frame, a chute guide device and a loading system. In consideration of the supporting engineering and laboratory conditions, the similarity ratio of the model is proposed to be 1:25. Therefore, the length, width and height of the model box are determined to be 5, 2 and 1.5 m, respectively. The model box adopts a modular design that can simulate the fault dip range of 45°–90° through module switching and enable the precise control of the sliding surface through the guiding function of the chute. The loading system adopts a numerically controlled synchronous hydraulic jacking device and is equipped with a computer digital servo control system, which can synchronously dislocate upward or downward to simulate forward and reverse fault dislocation. The control system regulates the speed of fault and the fault displacement by setting the flow rate and flow limits of the oil cylinder. The design of the fault dislocation simulation test device is shown in Fig. 4. Furthermore, ABAQUS finite element software is used to establish the numerical model of tunnel dislocation simulation, and the boundary effect of the model box is verified (the verification process is omitted from this paper due to space limitations).

### Similar materials and model making

In the model test, density, length, and gravitational acceleration are used as the basic physical quantities. The similarity ratios of various physical quantities are derived and shown in Table 2.

**Table 2:** Physical quantity similarity ratio in the model test.

Classification	Physical quantity	Dimension	Similarity relationship	Similarity ratio
Basic physical quantities	Length	L	$C_{L}$	1/25
	Density	FT^−2^ L^−4^	$C_{\rho }$	1/1.5
	Gravitational acceleration	LT^−2^	$C_{g}$	1
	Elastic modulus	FL^−2^	$C_{E} = C_{\rho } C_{g} C_{L}$	1/37.5
	Stress	FL^−2^	$C_{\sigma } = C_{\rho } C_{g} C_{L}$	1/37.5
	Strain	–	$C_{\varepsilon } = 1$	1
	Poisson ratio	–	$C_{\mu } = 1$	1
	Cohesion	–	$C_{c} = C_{\rho } C_{g} C_{L}$	1/37.5

#### Preparation of similar materials of surrounding rock

The physical and mechanical parameters of the rocks surrounding the hanging wall, foot wall and fault zone and their similar materials are determined in accordance with similarity theory and by relying on the surrounding rock conditions of the project^35^. These parameters are shown in Table 3.

**Table 3:** Physical and mechanical parameters of the surrounding rock and its similar materials.

Category		Density (kg/m^3^)	Elastic modulus (MPa)	Cohesion (kPa)	Internal friction angle (°)
Hanging wall and footwall	Prototype value	2400	1300	500	29
	Target value of similar materials	1600	34.67	13.33	29
Fault fracture zone	Prototype value	2200	500	100	24
	Target value of similar materials	1468	13.33	2.67	24

**Figure 5:**
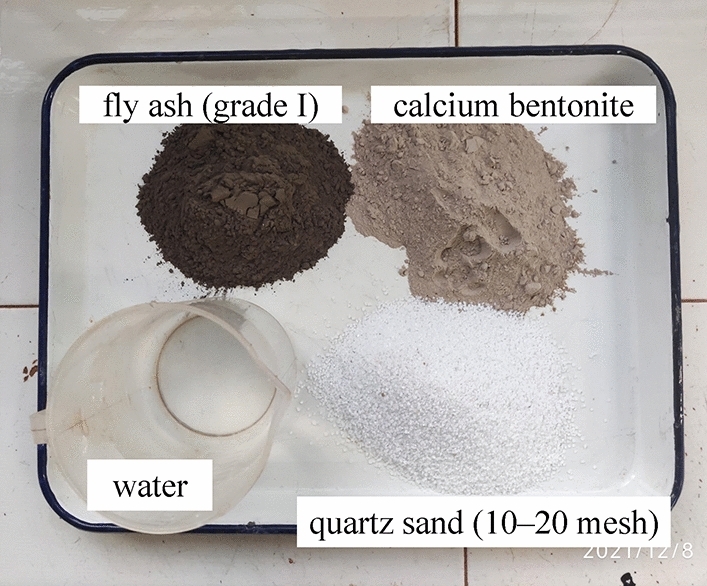
Composition of similar materials for surrounding rock.

**Figure 6:**
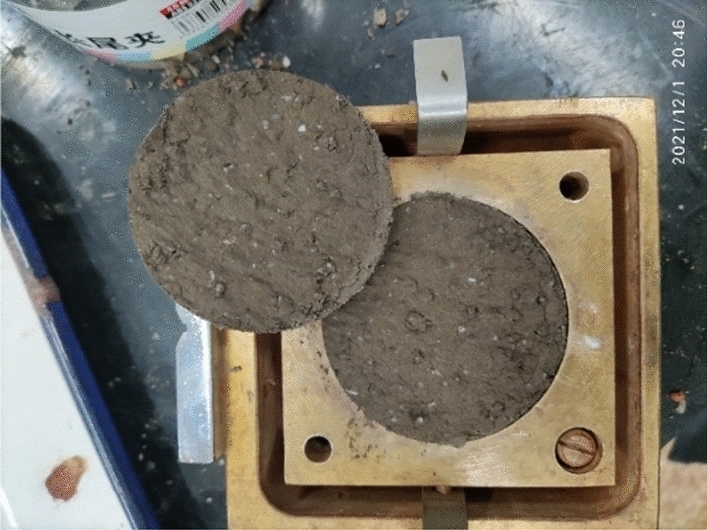
Shear test of similar materials for surrounding rock.

On the basis of the summary of previous research results, the research group used four raw materials, namely, fly ash (grade I), quartz sand (10–20 mesh), calcium bentonite and water to prepare the similar materials for the hanging wall and footwall rock. After a large number of proportioning tests, the weight proportion of the hanging wall and footwall rock similar materials is finally determined as fly ash : quartz sand : calcium bentonite : water = 1:0.4:0.2:0.54. The similar materials of the surrounding rock have the density of 1687 kg/m^3^; the elastic modulus of 35.72 MPa; the cohesion of 13.55 kPa and the internal friction angle of 30°, which is close to the target value of the similar materials of the surrounding rock and meets the required similarity ratio. The cohesion of the target value of similar materials in the fault fracture zone is low. Therefore, dry yellow sand is used to simplify the simulation of the surrounding rock of the fault zone. The similar materials in the fault zone have the density of 1550 kg/m^3^ and the elastic modulus of 17.43 MPa. The composition of similar materials for surrounding rock is shown in Fig. 5. The shear test of similar materials for surrounding rock is shown in Fig. 6.

#### Preparation of tunnel plaster model

##### Similar materials for C30 concrete

**Figure 7:**
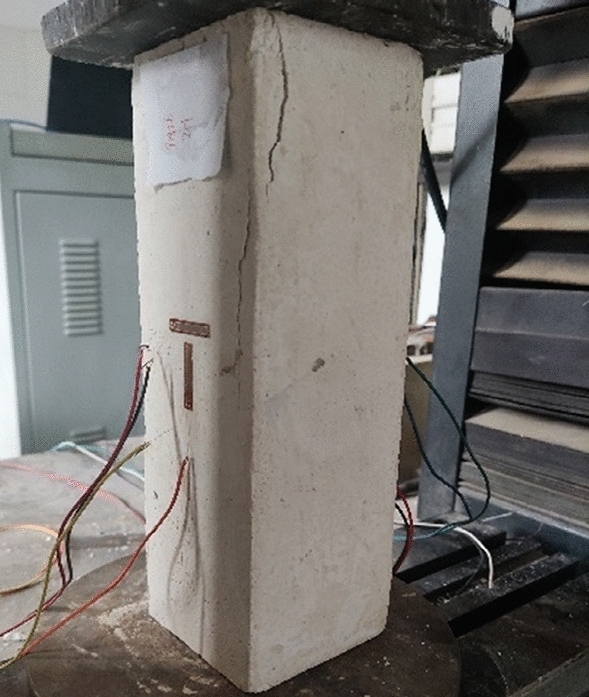
Uniaxial compression test.

**Figure 8:**
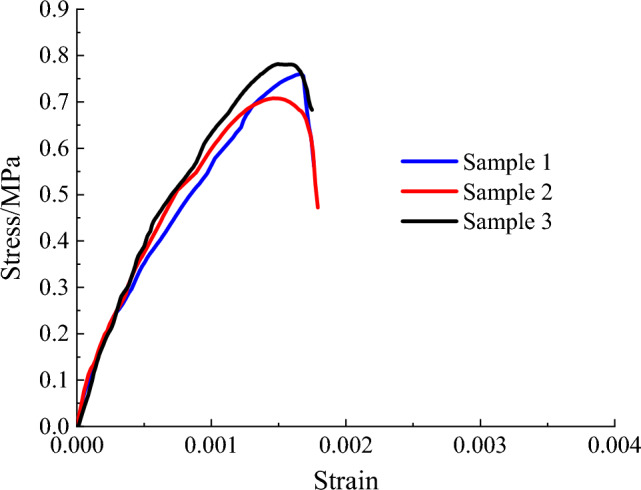
Stress–strain-damage evolution curve.

The main physical and mechanical parameters of C30 concrete and its similar materials are shown in Table 4^36^. The similar materials of C30 concrete are prepared with the basic materials of gypsum, barite powder, quartz sand and water. On the basis of a large number of proportioning tests, the elastic modulus and cube compressive strength of similar materials are measured through uniaxial compression test. The uniaxial compression test is shown in Fig. 7. The stress–strain-damage evolution curve is shown in Fig. 8. Then, the weight proportion of the similar materials of C30 concrete is determined as gypsum : barite powder: quartz sand : water = 1:1.80:1.40:1.48. The errors in material density and strength are 4.08% and 2.95%, respectively. The elastic modulus of the material is 766.90 MPa, with an error of 4.14% compared to the target value of 800 MPa. The average peak compressive strain is 0.154%, indicating that similar materials meets the mechanical parameter requirements. Comparing the results in Table 4 reveals that the difference between the experimental value and the target value of similar materials is small and thus meets similarity requirements.

**Table 4:** Physical and mechanical parameters of C30 concrete and its similar materials.

Category	Density (kg/m^3^)	Elastic modulus (GPa)	Cube compressive strength (MPa)	Peak compressive strain (%)
C30 concrete	2400	30	30	0.20
Target value of similar materials	1600	0.80	0.80	0.20
Test value of similar materials	1668	0.77	0.82	0.154

##### Similar materials of ECC

Most of the existing studies on similar materials of fibre-reinforced concrete added various fibres to microconcrete or gypsum mixtures^37^. Research on the similar materials of PVA-ECC has not been reported, but the existing studies have been noted to show that the combination of plastic concrete and PVA fibre can provide good ductility and toughness and that the compressive strength of the resulting material is low^38^. Therefore, this work uses this concept for reference and attempts to improve it to meet the test requirements.

**Figure 9:**
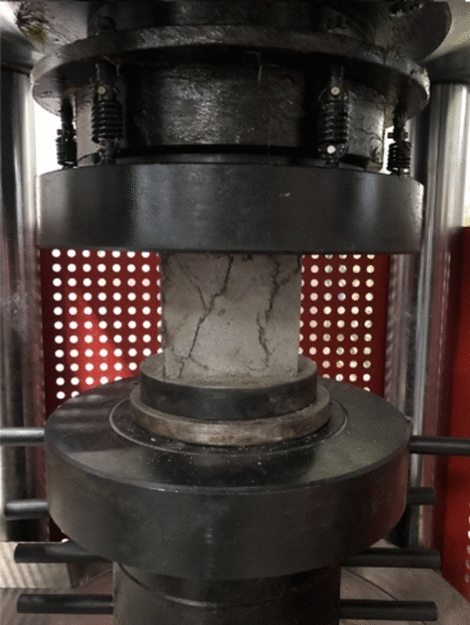
Uniaxial compression test.

**Figure 10:**
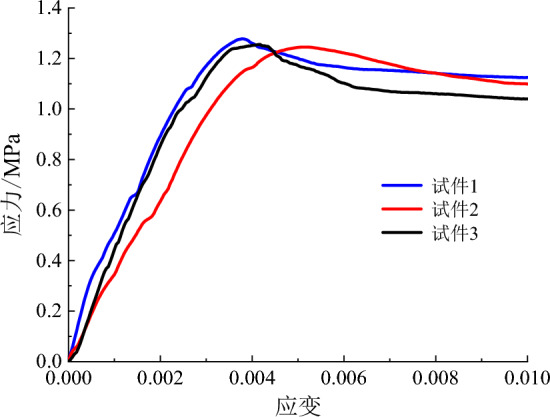
Stress–strain-damage evolution curve.

The uniaxial compression test is shown in Fig. 9. The stress–strain-damage evolution curve is shown in Fig. 10. Similarly, through a large number of material mechanical property tests, the proportion of PVA-ECC similar materials is identified as cement : gypsum : barite powder : quartz sand : sodium bentonite : water : PVA fibre = 5.3:19:19:24:2:30:0.7. The errors in material density and strength are 25.07% and 2.25%, respectively. The elastic modulus of the material is 454.57 MPa, with an error of 13.89% compared to the target value of 400MPa. The average peak compressive strain is 0.44%, indicating that similar materials meets the mechanical parameter requirements. Table 5 shows the main physical and mechanical parameters of the ECC prototype and its similar materials, in which the prototype parameters are obtained on the basis of previous research results. The table shows that the density of ECC similar materials is relatively high, but considering that making the model design parameters fully similar to the prototype material parameters is difficult, only the similarity of the main mechanical parameters can be met.

**Table 5:** Physical and mechanical parameters of ECC and its similar materials.

Category	Density (kg/m^3^)	Elastic modulus (GPa)	Cube compressive strength (MPa)	Peak compressive strain (%)
PVA-ECC	1900	15	41.8	0.45
Target value of similar materials	1267	0.40	1.11	0.45
Test value of similar materials	1690	0.45	1.14	0.44

The model lining reinforcement is simulated using iron wire, the process of calculating lining reinforcement according to the principle of strength equivalence is as follows:

1 $$ \frac{{A_{s} f_{y} }}{{A_{c} f_{c} }} = \frac{{A{}_{s}{\prime} f{}_{y}{\prime} }}{{A{}_{c}{\prime} f{}_{c}{\prime} }} $$

where *A*_*s*_ and *A*_*s*_*'* are the cross-sectional areas of the reinforcement in the prototype and the model, respectively; *f*_*y*_ and* f*_*y*_*'* are the strengths of the reinforcement in the prototype and the model, respectively; *A*_*c*_ and *A*_*c*_*'* are the cross-sectional areas of the concrete in the prototype and the model, respectively; *f*_*c*_ and *f*_*c*_*'* are the strengths of the concrete in the prototype and the model, respectively.

### The shock absorption layers and aseismatic joints

**Figure 11:**
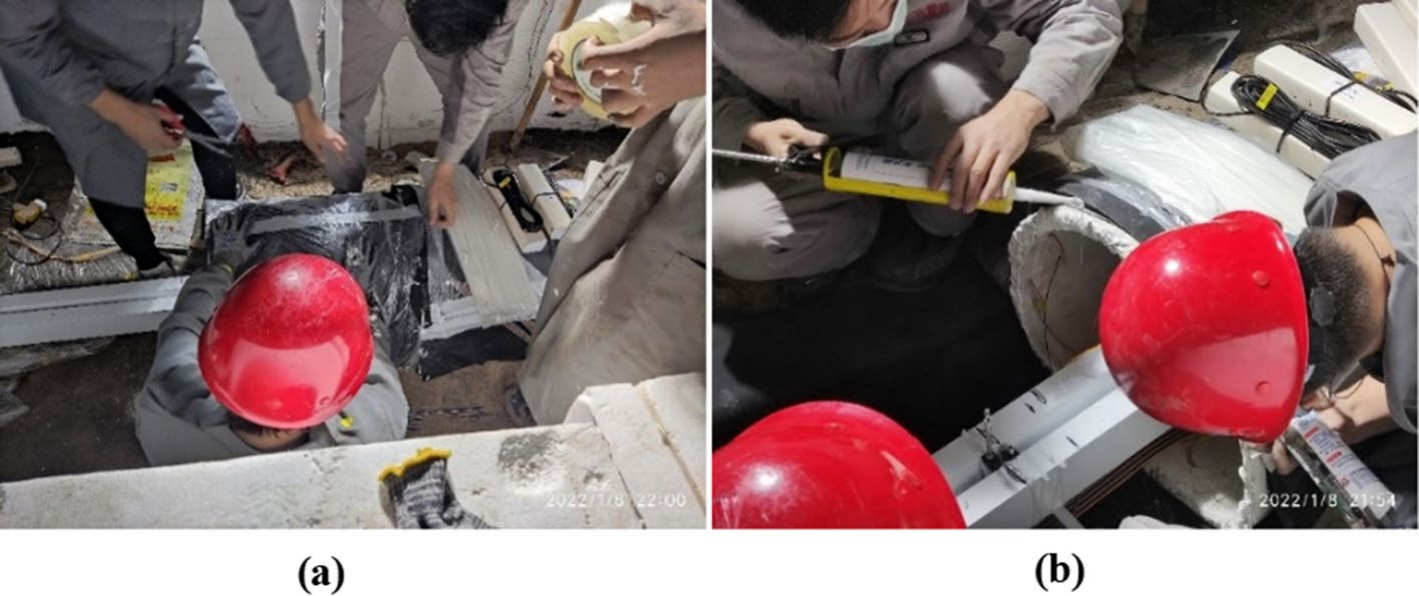
The shock absorption layers and aseismatic joints. (**a**) Shock absorption layer (**b**) Cementation at aseismatic joint.

In tunnel model tests, sponge rubber plates are often used to simulate the rubber shock absorption layer^39^ and cemented connections are usually used at aseismatic joints^40^. In this series of tests, sponge rubber plates with the thicknesses of 2 and 4 mm are used to simulate shock absorption layers with the thicknesses of 5 and 10 cm, respectively. Industrial silica gel adhesive is used to connect the lining sections, and adhesive tape is pasted outside the bonding place to simulate the damping joint. The setting of the shock absorption layers and aseismatic joints is shown in Fig. 11.

### Experimental content and sensor layout

**Figure 12:**
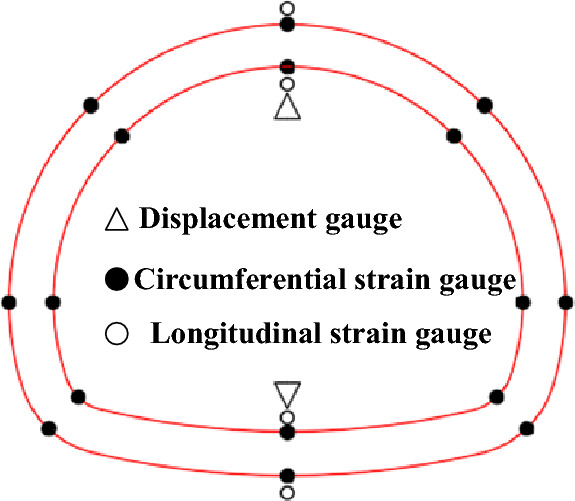
Layout of experimental elements.

In this test, the middle part of each lining segment is selected as the test section, and each test condition is set with 10 sections as shown in Fig. 3. In Fig. 3, serial numbers ① to ⑩ represent the number of lining segments, and serial numbers 1 to 10 represent the number of monitoring sections. Displacement gauge are arranged at the inner side of the crown and invert of each section. At the same time, longitudinal and circumferential strain gauges are arranged at the inner and outer crown, spandrel, side wall, arch springing and invert of each monitoring section. The test content mainly includes the strain and displacement of the structure. The layout of section measuring points and main sensors are shown in Fig. 12.

### Experimental steps and loading scheme

The main steps of this test are as follows: (1) Assemble and debug the fault dislocation simulation test system. (2) Prepare the similar materials of the surrounding rock and lining, process the lining mould, pour the lining model and paste the strain gauge at the measuring point after the formwork of the lining section is removed for maintenance. (3) Fill the prepared the similar materials of the surrounding rock into the model box layer by layer in accordance with the respective positions of the hanging wall, footwall and fault area; tamp and level every 20 cm filled; sample and weigh with a ring knife to check the compactness; roughen the filling surface after meeting the requirements and continue to fill to the reserved position of the tunnel. (4) Place the lining sections into the model box in turn; arrange the shock absorption layer and connect the lining sections and set the displacement meter, strain gauge and high-definition camera at the same time. Fill the surrounding rock material to the design ground height and carry out counterweighing. (5) Check the setting and performance of test elements and connect them to the acquisition instrument, debug the data acquisition instrument and set it to zero initially and complete the preparations before fault dislocation simulation. (6) Load in accordance with the displacement mode and take the dislocation displacement of the hanging wall (use *δ* to indicate the amount of dislocation) as the control object, load stepwise with increments of 1 cm and take the cumulative amount of dislocation *δ* = 9 cm as the test termination condition. Observe and measure the cracks and failure state of the tunnel structure during loading and after the test.

## Experimental results and discussion

### Displacement response

**Figure 13:**
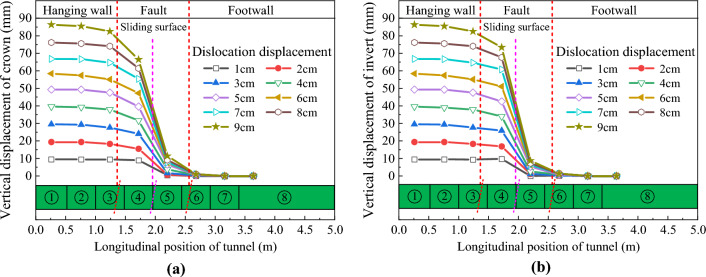
Vertical displacement distribution curve of CCSS. (**a**) Vertical displacement of the crown (**b**) Vertical displacement of the invert.

**Figure 14:**
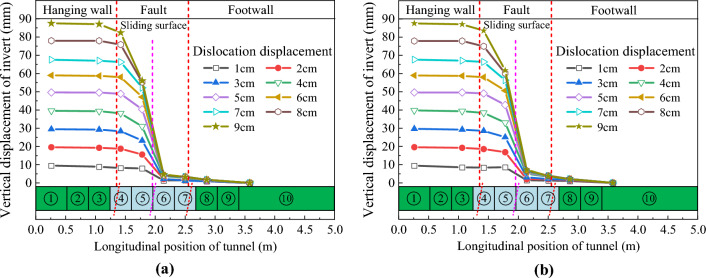
Vertical displacement distribution curve of ECSS. (**a**) Vertical displacement of the crown (**b**) Vertical displacement of the invert.

Figures 13 and 14 respectively show the vertical displacement distribution curves of the crowns and inverts of CCSS and ECSS under reverse fault dislocation. The figure revealed that the vertical displacement response laws of the two structures are basically the same. Under different dislocation displacements, the vertical displacements of the crowns and inverted arches of the two structures are distributed in a ‘S’ shape along the longitudinal direction. An obvious displacement difference is found between the lining sections in the area adjacent to the fault dislocation plane, and the maximum displacement difference is located at the fault dislocation plane. With the increase in the fault dislocation distance, the displacement difference between the lining segments on both sides of the fault dislocation plane increases nonlinearly, the amount and range of dislocation between the lining segments increase and the phenomenon of lining dislocation becomes increasingly obvious. Figure 13 shows that the vertical displacement difference of CCSS is mainly distributed within the range of 0.75–2.75 m along the longitudinal direction (equivalent to the 50 m range of the prototype, approximately 3.7d, where D is the tunnel span of the conventional structure), indicating that this section is the main section of the structure affected by fault dislocation. When current fault dislocation displacement *δ* = 9 cm (equivalent to the dislocation of the prototype structure of 2.25 m), the displacement differences (that is, the amount of dislocation) of the arch crown and inverted arch of the lining segments at the fault dislocation plane (④ and ⑤ lining segment in Fig. 2) reach 55.05mm and 63.63mm, respectively. Figure 14 shows that the dislocation of ECSS mainly affects the section within the range of 1–2.5 m (equivalent to the 37.5 m section of the prototype, approximately 2.7d), which has reduced by 14.3% compared with that of the conventional structure. When current fault dislocation displacement *δ* = 9 cm, the displacement differences of the arch crown and inverted arch between the ⑤ and ⑥ lining segments of ECSS are 51.40 and 62.85 mm, respectively, and the amount of dislocation between the lining segments of ECSS is lower than that of CCSS. ECSS weakens the disturbance of fault dislocation to the structure by shortening the spacing of aseismatic joints.

Under the reverse fault dislocation, the displacement is applied from the bottom of the upper plate of the model box. The inverted arch is affected before the arch crown. The damage to the tunnel develops from the inverted arch to the arch crown. Due to the compression of the rock and the deformation of the tunnel structure, part of the displacement effect is offset, resulting in a slightly larger displacement at the inverted arch than at the arch crown. The displacement direction can be considered as one of the factors affecting the design of defense measures.

**Figure 15:**
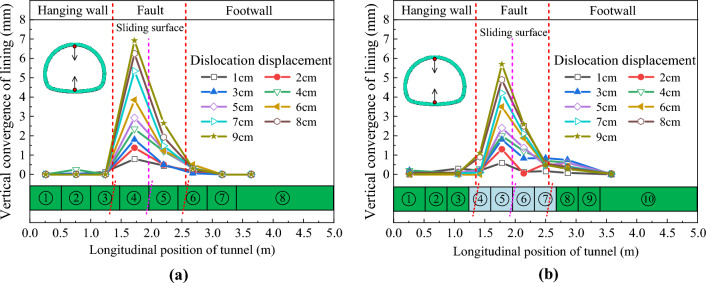
Distribution curve of the vertical convergence of the structure along the longitudinal direction. (**a**) Vertical convergence of CCSS, (**b**) Vertical convergence of ECSS.

The relative displacement results between the crowns and inverts of the tunnel structures are extracted, and the vertical convergence distribution curves of the two structures are obtained as shown in Fig. 15. Figure 15 illustrates that the vertical convergence distribution characteristics of the two structures are similar along the longitudinal direction. In particular, the vertical convergence deformation is mainly concentrated in the hanging wall and the footwall near the sliding surface. The vertical convergence value increases nonlinearly with the increase in fault dislocation displacement. The vertical deformation of the tunnel located in the rock mass of the hanging wall is significantly greater than that of the footwall. The vertical convergence value of ECSS under different dislocation displacements is smaller than that of CCSS. For example, when fault dislocation displacement δ = 9 cm, the maximum vertical convergence value of CCSS is 6.9 mm, whereas that of ECSS has decreased by 17.4% to 5.7 mm.

### Longitudinal deformation characteristics

**Figure 16:**
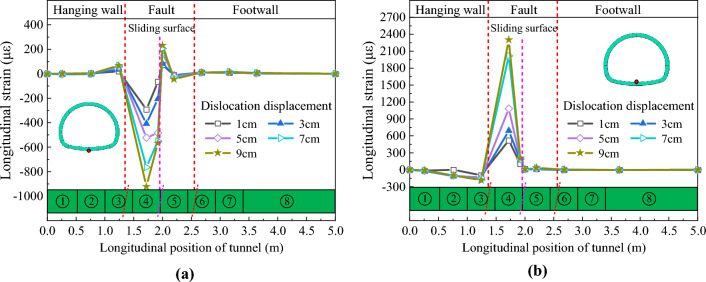
Longitudinal strain distribution curve of the invert of CCSS. (**a**) Outside of the invert, (**b**) Inside of the invert.

**Figure 17:**
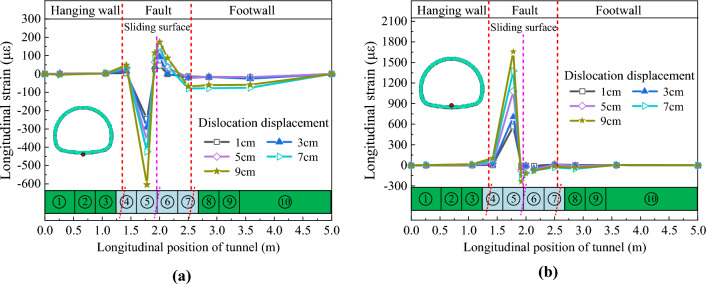
Longitudinal strain distribution curve of the invert of ECSS. (**a**) Outside of the invert, (**b**) Inside of the invert.

**Figure 18:**
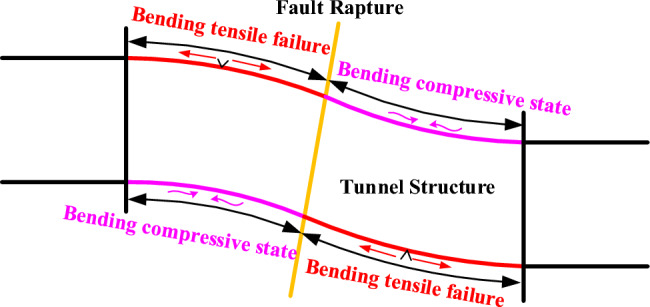
Schematic diagram of the longitudinal tension bending damage mode of the tunnel lining.

The invert longitudinal strain is taken as an example. Figures 16 and 17 respectively show the distribution curves of the longitudinal strain of the inverts of CCSS and ECSS along the longitudinal direction of the tunnel. In the figures, positive strain indicates tension, and negative strain indicates compression. The figure reveals that the longitudinal strain of the inverts of the two structures is mainly concentrated in the fault sliding surface and hanging wall area. Under the same dislocation displacement, the longitudinal strain of the tunnel invert within the hanging wall shows that the outer side is under pressure and the inner side is under tension, whereas the longitudinal strain of the tunnel invert within the footwall is less affected by the dislocation. Considering the longitudinal bending deformation characteristics of the tunnel structure, the damage mode of the longitudinal tensile strain overrun area is considered to be tensile bending damage, and its damage mode is shown in Fig. 18.

When the amount of dislocation *δ* = 9 cm, the tensile and compressive strain peaks of CCSS invert reach 2302.8 and 921.2 με, respectively, and the extreme tensile strain value of the lining material is exceeded, resulting in longitudinal cracking failure at the invert of lining segments ④ and ⑤ in the fault fracture zone. Under the same dislocation displacement, the tensile and compressive strain peaks of the invert of ECSS are 1656.2 and 604.8 με, respectively, and have reduced by 28.1% and 34.3%, respectively, relative to those of CCSS. At this time, only small cracks are distributed on the inner surface of the invert of the lining segment in the dislocation affected area of ECSS, and no through cracks appear. These results show that by improving the material properties and increasing the thickness of the shock absorption layer, the stress conditions of the lining are improved and the adaptability of the structure to deformation is enhanced.

### Circumferential deformation characteristics

**Figure 19:**
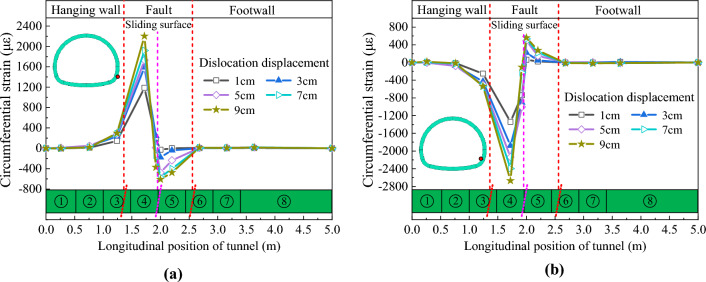
Circumferential strain distribution curve of the arch springing of CCSS. (**a**) Outside of the arch springing, (**b**) Inside of the arch springing.

**Figure 20:**
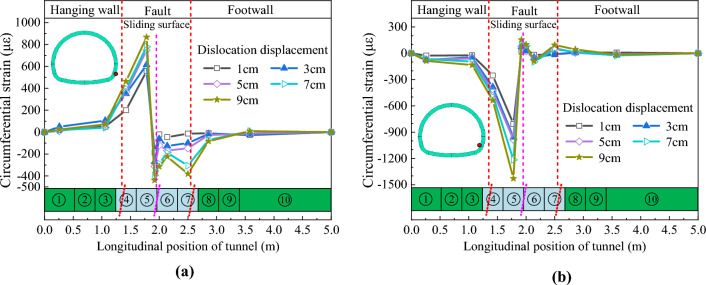
Circumferential strain distribution curve of the arch springing of ECSS. (**a**) Outside of the arch springing, (**b**) Inside of the arch springing.

Figures 19 and 20 depict the circumferential strain distribution curves of the arch springing of CCSS and ECSS, respectively. Figures 19 and 20 show that under different dislocation amounts, the circumferential strains of the arch springing of the two structures change sharply near the sliding surface and decrease rapidly with the increase in the distance from the sliding surface. For CCSS, the tensile and compressive strain changes at the arch springing are concentrated in the section of 0.75–2.75 m (approximately 3.7D). The strain variation of ECSS is concentrated in the section of 1.0–2.75 m (approximately 3.2D). The maximum arch springing strain of the two structures increases nonlinearly with the increase in fault displacement. Compared with those of CCSS, the tensile and compressive strain peaks of ECSS are significantly reduced under the same dislocation displacement. For example, when fault dislocation displacement *δ* = 9 cm, the maximum tensile and compressive strains of the arch springing of CCSS are 2202.2 and 2669.2 με, respectively, and those of ECSS have decreased by 60.6% and 46.5% to 866.9 and 1428.8 με, respectively.

**Figure 21:**
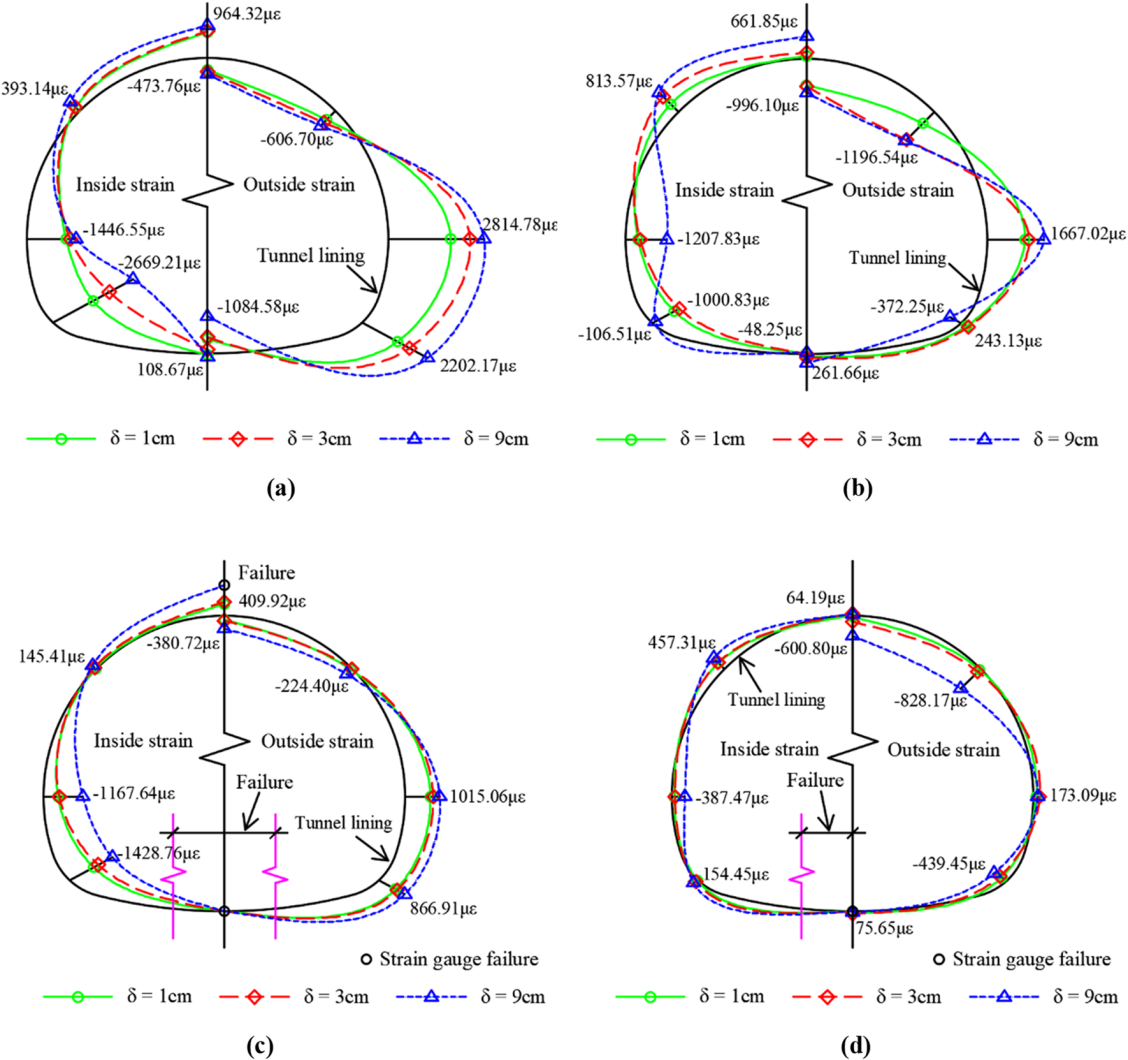
Circumferential strain distribution of typical lining section. (**a**) No. 4 monitoring section of CCSS, (**b**) No. 5 monitoring section of CCSS, (**c**) No. 4 monitoring section of ECSS, (**d**) No. 5 monitoring section of ECSS.

The above analysis illustrates that the tunnel structure in the fracture zone near the sliding surface and hanging wall area are more obviously affected by fault dislocation than other structures. Consequently, the typical dislocation displacement (*δ* = 1, 3, 9 cm) and the circumferential strain distribution diagrams of the inner and outer surfaces of the monitoring sections No. 4 and No. 5 of the tunnel structure are plotted in Fig. 21. Figure 21 shows that the circumferential strain distribution laws of the two support structures are similar and manifest as follows: As the inside strain of the crown and spandrel is tensioned, the outside strain is compressed, the inside strain of the side wall and the arch springing is compressed and the outside strain is tensioned, that is, the lining cross-section shows the characteristics of vertical inward compression deformation and horizontal passive outward deformation. With the increase in fault dislocation displacement, the strains of the side wall and arch springing of the two supporting structures increase significantly, and the maximum strains are located at the No.4 monitoring section. When the displacement of the fault dislocation is increased from 3 to 9 cm, the strain of CCSS decreases and the strain changes from tension to compression on the inside and outside of the arch springing at the No. 5 monitoring section due to local collapse and dislocation near this part, resulting in the rebound of the structural strain. With the increase in fault dislocation displacement, the overall strain value of ECSS becomes smaller than that of CCSS, and lining collapse does not occur near the sliding surface.

### Structural cracking and failure characteristics

**Figure 22:**
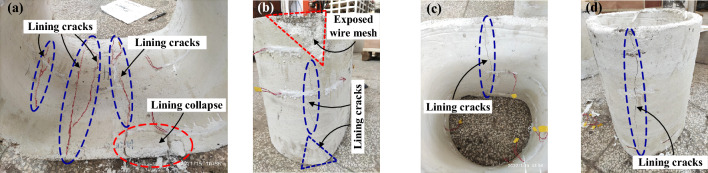
Failure mode of CCSS. (**a**) Invert of segment④ (**b**) Arch springing of segment④ (**c**) Invert of segment⑤ (**d**) Arch springing of segment⑤.

**Figure 23:**
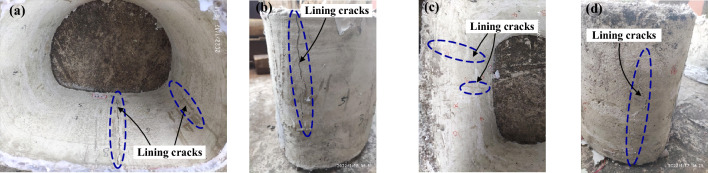
Failure mode of ECSS. (**a**) Invert of segment⑤ (**b**) Arch springing of segment⑤ (**c**) Invert of segment⑥ (**d**) Arch springing of segment⑥.

The crack distribution, lining deformation and typical failure characteristics of CCSS and ECSS are illustrated in Figs. 22 and 23. Under the concentrated dislocation displacement mode, the failure modes of the two structures are the same and are characterised by lining dislocation, cracking and failure localisation. Given that the aseismatic joints are set between the lining segments, the damage of the two structures under the forced dislocation of the fault is concentrated in the fault fracture zone and the hanging wall area near the sliding surface.

The damage of CCSS under reverse fault dislocation is concentrated in the ③, ④ and ⑤ segments on both sides of the sliding surface, that is, within 1.0–2.44 m (2.6D) along the longitudinal direction of the tunnel, and the damage is mainly located at the arch springing, near the side wall and the invert. The lining cracks are mainly longitudinal cracks, and most are internal and external through cracks. Amongst the segments, the ④ lining segment is the most severely damaged and the inside of the invert and the outside of arch springing of this segment have fallen off the lining and exposed steel mesh. The damage to ECSS under reverse fault dislocation is concentrated in the ⑤ and ⑥ segments on both sides of the sliding surface, that is, within the range of 1.60–2.32 m (1.3D) along the longitudinal direction of the tunnel, further reducing the damage distribution range of ECSS compared with that of CCSS. The cracking of the ⑤ segment in the hanging wall is relatively obvious and the cracking of the ⑥ segment in the footwall is mostly in the form of microcracks and lacks obvious damage. Although the cracking position of ECSS is roughly the same as that of CCSS, the cracking degree of ECSS is significantly reduced, and the absence of internal and external through cracks and local spalling demonstrate the excellent crack control ability and obvious antispalling effect of ECSS.

To summarise, by shortening the spacing of aseismatic joints and increasing the thickness of the shock absorption layer and by benefitting from the crack resistance and toughening effect of PVA fibres, ECSS significantly improves the stress conditions of the structure, enhances the anti-dislocation of the tunnel and shows improved anti-dislocation applicability.

## Finite element analysis

### Finite element model

**Figure 24:**
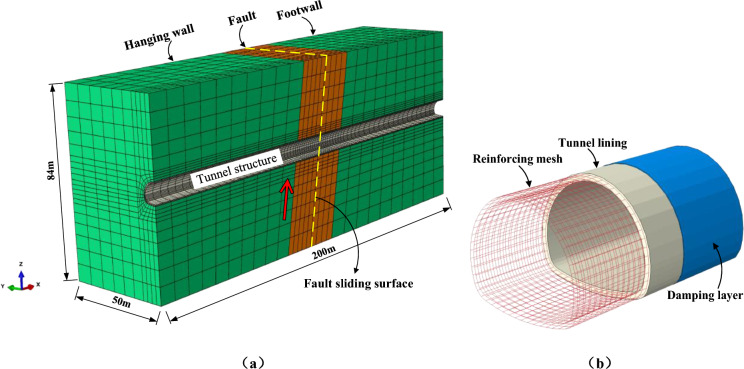
Finite element model to simulate tunnel dislocation across active fault. (**a**) Mesh of the overall model, (**b**) Supporting structure.

According to the design parameters of the supporting tunnel, a three-dimensional numerical model of fault dislocation is established for two composite support structures, taking into account the interaction of the surrounding rock-fault-tunnel structure. The finite element model of typical conditions is shown in Fig. 24. The design parameters such as the length of the lining segment, the spacing of the aseismic joints and the thickness of the damping layer are taken according to the tunnel project. The fault fracture zone is 30 m wide, with the dip angle of 80°. The length of the model is 200 m, the width of the model is 100 m, the height of the model is 84 m.

In the model, the rock mass is assumed to be an elastic-perfectly plastic material, and the Mohr–Coulomb yield criterion is adopted for the constitutive model of the rock mass. The interaction between the surrounding rock and the tunnel lining is achieved by the application of contact pairs in ABAQUS to simulate the relative deformation and contact force transmission during fault dislocation. The Coulomb friction law using the penalty method is adopted to simulate the tangential behaviour of the interface. Table 6 lists the physical and mechanical properties of the materials used in the model^41^.

**Table 6:** Physical and mechanical parameters of surrounding rock and damping layer.

Category	Elastic Modulus /(GPa)	Density /(kg/m^3^)	Cohesion /(MPa)	Poisson's ratio	Internal friction angle /(°)
Hanging wall and footwall	2	2400	0.5	0.34	29
Fault	0.5	2200	0.1	0.40	24
Shock absorption layer	0.2	1000	-	0.38	-

Grade-C30 concrete and the ECC materials are used for the secondary lining, and the grade is consistent with the Chinese Code for design of concrete structures (GB 50,010–2010). The Concrete Plastic Damage (CDP) model is adopted for the constitutive model of the Grade-C30 concrete and the ECC. Table 7 lists the physical and mechanical properties of the two materials.

**Table 7:** Physical and mechanical properties of C30 concrete and the ECC.

Material	experiment method	Yield stress (MPa)	Yield stress/%	Peak stress (MPa)	Peak strain/%	Ultimate stress (MPa)	Ultimate strain/%	Elastic modulus (GPa)	Poisson's ratio	Density (kg/m^3^)
C30 concrete	Uniaxial tension	1.54	0.0051	2.89	0.01	0.18	0.19	30	0.2	2500
	Uniaxial compression	12	0.04	30.0	0.15	2.0	2.0			
ECC	Uniaxial tension	3.2	0.021	3.67	3.27	–	7.0	15	0.2	1900
	Uniaxial compression	32.2	0.18	41.8	0.45	–	4.5			

The ECC uniaxial tensile stress–strain relationship can be expressed as follows^42^:

2 $$ \sigma_{t} = \left\{ {\begin{array}{*{20}c} {\frac{{\sigma_{t0} }}{{\varepsilon_{t0} }}\varepsilon_{t} } & {0 \le \varepsilon_{t} < \varepsilon_{t0} } \\ {\frac{{\sigma_{tp} - \sigma_{t0} }}{{\varepsilon_{tp} - \varepsilon_{t0} }}\left( {\varepsilon - \varepsilon_{t0} } \right) + \sigma_{t0} } & {\varepsilon_{t0} \le \varepsilon_{t} < \varepsilon_{tp} } \\ {\frac{{\sigma_{tp} \left( {\varepsilon_{tu} - \varepsilon_{t} } \right)}}{{\varepsilon_{tu} - \varepsilon_{tp} }}} & {\varepsilon_{tp} \le \varepsilon_{t} < \varepsilon_{tu} } \\ 0 & {\varepsilon_{tu} \le \varepsilon_{t} } \\ \end{array} } \right. $$

where *σ*_t0_ and *ε*_t0_ are ECC uniaxial tensile yield stress and yield strain respectively; *σ*_tp_ and *ε*_tp_ are are ECC uniaxial tensile peak stress and strain corresponding to the peak stress; *σ*_tu_ and *ε*_tu_ is ECC uniaxial tensile ultimate stress and strain corresponding to the ultimate stress.

The ECC uniaxial compressive stress–strain relationship can be expressed as follows^43^:

3 $$ \sigma_{c} = \left\{ {\begin{array}{*{20}c} {E_{c0} \varepsilon_{c} } & {0 < \varepsilon_{c} < \varepsilon_{c0.4} } \\ {E_{c0} \varepsilon_{c} \left( {1 - 0.39\frac{{\varepsilon E_{c0} }}{{\sigma_{cp} }} + 0.25} \right)} & {\varepsilon_{c0.4} < \varepsilon_{c} < \varepsilon_{cp} } \\ { - 9470.6\left( {\varepsilon_{c} - \varepsilon_{cp} } \right) + \sigma_{cp} } & {\varepsilon_{cp} < \varepsilon_{c} < \varepsilon_{cl} } \\ { - 375.4\left( {\varepsilon_{c} - \varepsilon_{cl} } \right) + \sigma_{cl} } & {\varepsilon_{cl} < \varepsilon_{c} < \varepsilon_{cu} } \\ \end{array} } \right. $$

Where *E*_*c*0_ is the initial elastic modulus of ECC; *ε*_c0.4_ is the corresponding strain value when the ECC stress reaches 40% of the ultimate strength; *σ*_cp_ and *ε*_cp_ are the peak stress value of the ECC uniaxial compression and its corresponding strain value; *σ*_cl_ and *ε*_*c*l_ are the stress value and strain value at the inflection point of the ECC compression stress–strain curve respectively; *σ*_cu_ and *ε*_cu_ are the ultimate stress value of the ECC uniaxial compression and its corresponding strain value.

According to the Sidoroff energy equivalence principle^44^, the damage factor in the CDP model can be expressed as follows:

4 $$ d_{k} = 1 - \left( {\frac{{\sigma_{k} }}{{E_{0} \varepsilon_{k} }}} \right)^{0.5} , $$

Where *k* = *c* represents compression, *k* = *t* represents tension; *d*_*k*_ is the damage factor; *E*_0_ is the initial elastic modulus of the ECC; *σ*_*k*_ is the stress of the ECC; *ε*_*k*_ is the strain of the ECC.

**Figure 25:**
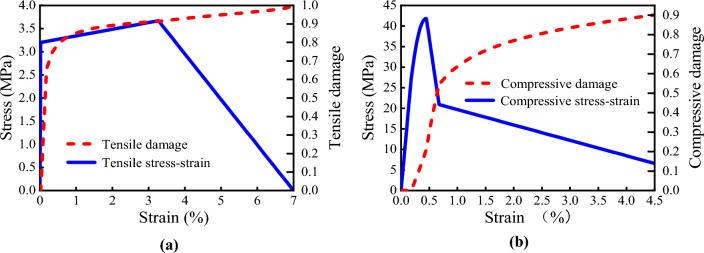
Stress–strain-damage evolution curve of the ECC. (**a**) Stress–strain-tensile damage, (**b**) Stress–strain-compression damage.

Based on the ECC experimental results of the mechanical properties carried out and Eqs. (1), (2) and (3), the obtained stress–strain-damage evolution curves of the ECC are shown in Fig. 25. Table 7 lists the physical and mechanical properties of the ECC material.

The elastic–plastic truss element is adopted to simulate the lining reinforcement. Table 8 lists the physical and mechanical properties of the reinforcement material. The interaction between the lining segments is taken into account, a limited sliding hard contact surface with a coefficient of friction of 0.2 is set on both sides of aseismic joint^28^.

**Table 8:** Physical and mechanical properties of reinforcement.

Elastic modulus /(GPa)	Poisson's ratio	Yield Stress /(MPa)	Yield strain /%	Ultimate stress /(MPa)	Ultimate tensile strain /%
200	0.3	400	0.2	540	7.5

### Model verification

**Figure 26:**
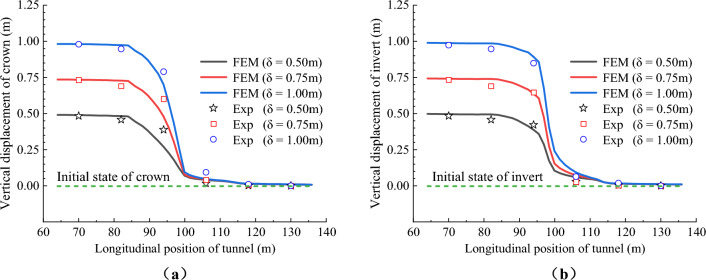
Comparison of experimental and numerical results of vertical displacement of the CCSS. (**a**) Vertical displacement of the crown, (**b**) Vertical displacement of the invert.

**Figure 27:**
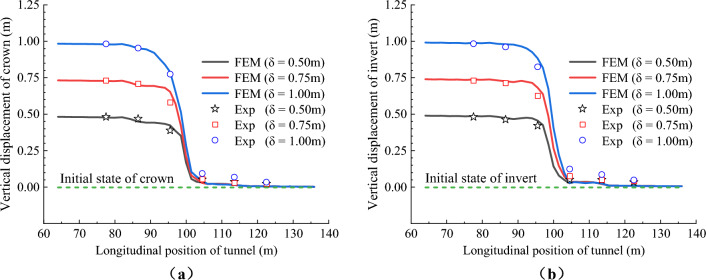
Comparison of experimental and numerical results of vertical displacement of the ECSS. (**a**) Vertical displacement of the crown, (**b**) Vertical displacement of the invert.

To verify the extent of reduction of the prototype through model testing, the vertical displacement data of six lining segments on both sides of the sliding surface were extracted. The displacement results from the model test were amplified by 25 times based on the geometric similarity ratio, and these amplified results were compared with the numerical calculation results. The comparison results of the vertical displacement of the two structures are shown in Figs. 26 and 27, respectively. Under dislocation displacement of 0.5m to 1.0 m, the vertical displacements of the crowns and inverted arches of the two structures are distributed in a ‘S’ shape along the longitudinal direction. The calculated displacement results closely match the experimental results. This indicates that the numerical simulation accurately represents the forced displacement mode of the tunnel under reverse fault dislocation.

### The dislocated response of the ECSS

The anti-dislocation performance of the ECSS under reverse fault dislocation is superior. To investigate the applicability of the ECSS under fault dislocation, the dislocated response of the ECSS was further analyzed based on the finite element model with surrounding rock conditions and different types fault dislocation. Table 9 lists the calculation cases. Table 3 lists the physical and mechanical properties of grade-IV and grade-V surrounding rock. For the Grade-VI surrounding rock, the density is 1800kg/m^3^, the elastic modulus is 200MPa, the poisson's ratio is 0.42, the internal friction angle is 20°, and the cohesion is 5kPa.

**Table 9:** Calculation cases.

Cases	Surrounding rock grade of hanging wall and foot wall	Surrounding rock grade of fault zone	Fault type (dislocation mode)
1	IV	V	Reverse fault
2	IV	VI	Reverse fault
3	V	VI	Reverse fault
4	IV	V	Normal fault
5	IV	V	Strike slip fault

#### Influence of surrounding rock conditions

**Figure 28:**
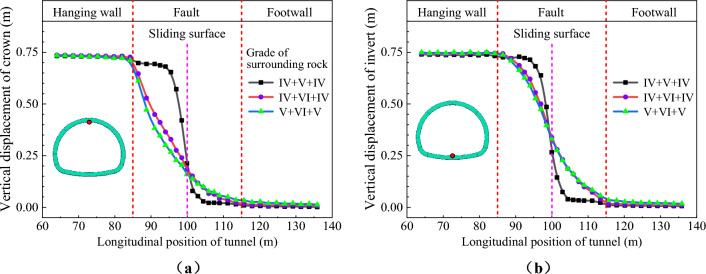
Structural displacement distribution curve under different surrounding rock conditions. (**a**) Vertical displacement of crown, (**b**) Vertical displacement of invert.

Figure 28 show the vertical displacement distribution curves of the ECSS under different surrounding rock conditions. The quality of the hanging wall, foot wall, and fault fracture zone significantly influence the fault dislocation of the structure. The distribution shape and range of vertical displacement is changed by changing the quality of surrounding rock. In the case of a fault dislocation of 0.75 m, under the calculation case 1, the vertical displacement distribution of the structure is mainly concentrated within a length range of approximately 18m on both sides of the sliding surface. However, under the calculation case 2 and 3, this range increased to about 30 m. Nevertheless, the slope of the displacement distribution curve in these sections are significantly reduced. This phenomenon indicates that the disturbance range under fault dislocation is increased and the degree of structural deformation is decreased by reducing the quality of surrounding rock. The restraint effect of surrounding rock on tunnel is decreased by reduced the quality of surrounding rock, which results in the range of structural deformation increased but the degree of deformation decreased.

**Figure 29:**
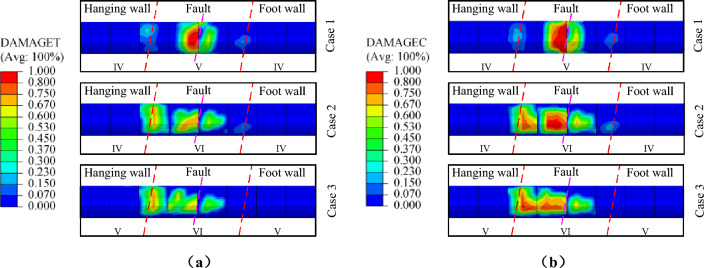
Damage distribution of lining structure. (**a**) Tensile damage,(**b**) Compressive damage.

When the fault dislocation displacement is 0.75m, the distribution cloud map of structural tensile and compressive damage is shown in Fig. 29. The results in the figure indicate that the structural tensile and compressive damage peaks of the case 1 are 0.823 and 0.901, respectively, which are significantly larger than those of case 2 and case 3. This phenomenon indicates that the damage degree of the structure is decreased by reduced the quality of surrounding rock in fault fracture zone. This difference can be attributed to the lower strength of the surrounding rock in the fault zone, which leads to greater deformation of the surrounding rock when squeezed by the fault dislocation. However, the damage range of the lining segment is significantly increased by decreased the quality of the surrounding rock in the fault zone. This implied that the difference in the amount of squeezing deformation under fault dislocation is increased by increasing the quality gap of surrounding rock between the hanging wall and the fault fracture zone. This can be likened to the structure at the junction of the hanging wall, foot wall and the fault fracture zone experienced an additional fault dislocation. Compared the case 2 and case 3, it can be observed that when the surrounding rock quality of the fault zone is poor (grade-VI), the structural damage extends to the junction of the hanging wall and the fault zone as the quality of the surrounding rock in the hanging wall and foot wall decreased. However, the overall degree and range of structural damage remain relatively smaller changed.

#### Influence of the types of fault dislocation

**Figure 30:**
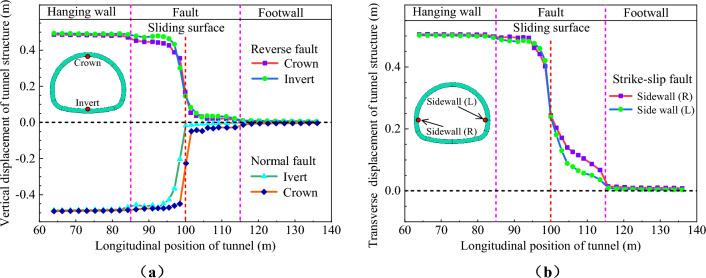
Displacement curve of structure under different types of fault dislocation. (**a**) Vertical displacement of structure (normal fault, reverse fault), (**b**) Horizontal displacement of structure (strike-slip fault).

Figure 30 show the vertical displacement distribution curves of the ECSS under different types of fault dislocation. For the normal and reverse fault dislocation, extracted the vertical displacement results of the structural crown and invert. For the strike-slip fault, extracted the horizontal displacement of the side walls on both sides of the structure. From Fig. 30, it is evident that the vertical displacement of the structure is distributed in a ‘S’ shape along the longitudinal direction under three types of fault dislocation, but the distribution ranges and characteristics are varied. The vertical displacement of the structure under normal and reverse fault dislocation is primarily concentrated in the hanging wall and the fault fractured zone near the hanging wall. However, the vertical displacement of the structure is distributed throughout the entire fault fractured zone under normal fault dislocation, with a range approximately twice of the reverse fault dislocation, and the vertical convergence of the structure is also significantly larger than normal fault dislocation. The horizontal displacement of the left and right walls of the structure under the strike-slip fault dislocation is mainly distributed in the hanging wall and the entire fault fracture zone, with a range significantly larger than normal and reverse fault dislocation. The horizontal convergence of the left and right walls of the structure is primarily distributed in the fault fracture zone near the foot wall. It is evident that the response law of the structural dislocation displacement varied depending on the type of fault dislocation.

**Figure 31:**
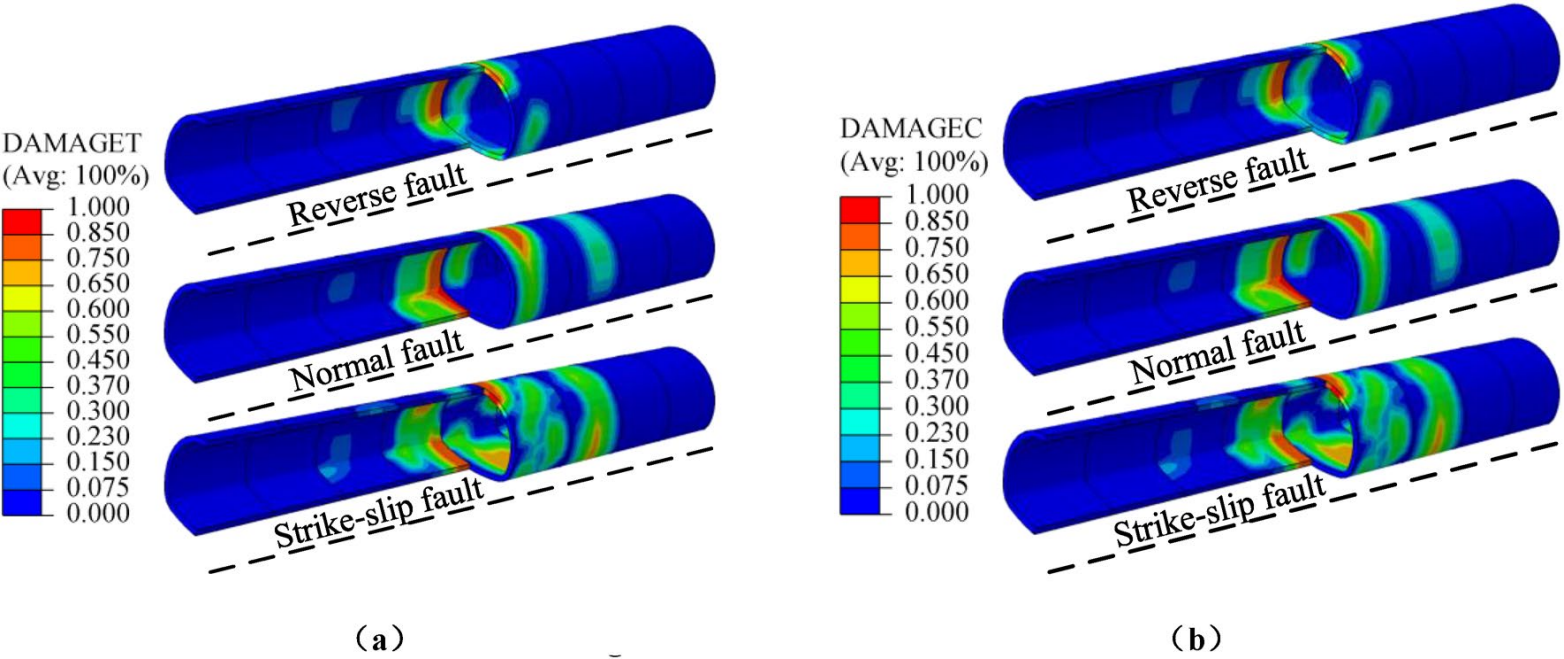
Distribution of structural damage under different types of fault dislocation. (**a**) Tensile damage, (**b**) Compressive damage.

When the fault dislocation displacement is 0.5m, the distribution cloud map of structural tensile and compressive damage under normal fault, reverse fault, and strike-slip fault dislocation is shown in Fig. 31. For the range of structural damage, the tensile damage range caused by three types of fault dislocation follows the order of strike-slip fault, reverse fault and normal fault from large to small, the compressive damage range caused by three types of fault dislocation follows the order of strike-slip fault, normal fault and reverse fault from large to small. For the degree of structural damage, the compressive damage value of the structure is similar under different types of fault dislocation, while the tensile damage value is highest under normal fault dislocation. This indicated that the damage degree of the structure is maximum under normal fault dislocation, and the damage range of the structure is widest under strike-slip fault dislocation,.

## Conclusions

The dislocation response model tests of ECSS and CCSS are carried out and the dislocation response characteristics of the two composite structures are discussed in this paper. Combined with the model tests and numerical analysis, the following conclusions can be drawn:

1. Under reverse fault dislocation, the vertical displacement of the two structures is distributed in a ‘S’ shape along the longitudinal direction of the tunnel. As the fault dislocation increases, the vertical convergence value of the structure and the amount and range of dislocation between lining segments increase non-linearly. In contrast to CCSS, the area affected is significantly decreased by fault dislocation of the ECSS.
2. The ECSS and the CCSS have consistent deformation patterns under reverse dislocation. The Longitudinal deformation of the two structures are concentrated in the fault fracture zone, with the strain peaks located near the sliding surface. Circumferential deformation characteristics are vertical inward extrusion deformation and horizontal passive outward deformation. In contrast to the CCSS, the ECSS ameliorates the stress conditions of the structure and enhances the adaptability of the structure to deformation by improving the material properties and increasing the thickness of the shock absorption layer.
3. In the concentrated dislocation displacement mode, the failure modes of the two structures are characterized by lining dislocation, cracking and localized failure. The ECSS benefits from the crack resistance and toughening effect of fibres. The degree and scope of cracking of ECSS are significantly reduced compared with those of the CCSS, and internal and external through cracks and local spalling are absent.
4. The overall damage degree of the ECSS is decreased and the damage range is increased by decreasing the strength of the surrounding rock in the fault zone. The local damage of the ECSS in fault zone near the hanging wall is increased and overall range and distribution of damage are little changed by decreasing the strength of the surrounding rock in the hanging wall, foot wall and fault zone.
5. The fault dislocation response pattern of the ECSS varies depending on the fault type. The damage degree caused by different types of fault dislocation follows the order of normal fault, strike-slip fault, and reverse fault from large to small. However, the damage range caused by the strike-slip fault is significantly larger compared to normal fault and reverse fault.

## Data Availability

The datasets generated during and analysed during the current study are available from the corresponding author on reasonable request.
